# Evolutionary Response Patterns of the Rodent *TLR* Genes to Climatic Factors

**DOI:** 10.1002/ece3.74271

**Published:** 2026-09-01

**Authors:** Chao Zhao, Tian Xia, Guangshuai Liu, Zhao Liu, Xinglei Ding, Shuo Dai, Honghai Zhang

**Affiliations:** ^1^ School of Life Sciences Qufu Normal University Qufu Shandong Province China; ^2^ Shandong Key Laboratory of Wetland Ecology and Biodiversity Conservation in the Lower Yellow River Qufu Shandong Province China

**Keywords:** climate factor, correlation, evolutionary rate, rodent, *TLR*

## Abstract

Toll‐like receptors (TLRs) are evolutionarily conserved pattern recognition receptors that play a crucial role in the innate immune response of animals. Their evolutionary dynamics are closely linked to pathogen pressure and environmental factors. At present, the relationship between the evolution of *TLR* genes and climatic factors remains poorly understood. In this study, we identified *TLR1*–*TLR9* genes from 42 rodent species with relatively restricted distribution ranges. Phylogenetic comparative analyses, including phyANOVA and PGLS, were conducted to assess the relationship between evolutionary rates (*ω* values) and 10 climatic factors. The results indicated that different *TLR* genes exhibit distinct climate‐associated patterns. Notably, increased precipitation‐related factors were positively associated with the evolutionary rates of *TLR2* and *TLR4*, whereas temperature‐related factors (Mean Diurnal Range) were positively correlated with the evolutionary rates of *TLR8* and *TLR9*. We hypothesized that both precipitation‐ and temperature‐related factors may indirectly influence the evolution of *TLR* genes by affecting the diversity and community composition of environmental pathogens. These results may enhance our understanding of the evolution of immune genes in animals through the perspective of potential ecological associations.

## Graphical abstract

This study systematically investigates the associations between climatic factors and the evolutionary dynamics of Toll‐like receptor (TLR) genes across 42 rodent species. Our findings demonstrate that temperature‐related climatic variables (Annual Mean Temperature, Mean Diurnal Range, and Max Temperature of Warmest Month) may modulate the evolutionary rates of TLR1, TLR6, TLR8, and TLR9, while precipitation‐related factors (Precipitation of Wettest Month and Precipitation of Coldest Quarter) primarily influence the evolutionary rates of TLR2 and TLR4. Collectively, these results offer novel insights into the ecological drivers underlying the adaptive evolution of immune genes.

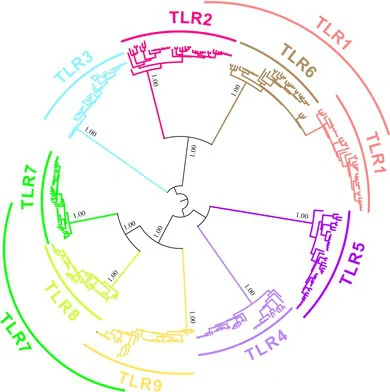

## 1 Introduction

Animals inhabiting diverse ecological niches encounter a variety of pathogens and environmental pressures, with both ecological and genetic factors influencing the prevalence and intensity of infection with pathogens in wild hosts (Civitello et al. 2015; Cornetti et al. 2018; Tschirren et al. 2013). Adaptation to complex environments in which pathogen communities vary is an important aspect of ecological adaptation in animals (Barreiro et al. 2009; Liu et al. 2020; Zhang et al. 2019). Among various vertebrate groups, the strength of host‐pathogen interactions may vary with latitude, leading to increased pathogen loads and disease incidence as one approaches the equator (Schemske et al. 2009). Climatic factors, including temperature and precipitation, have been demonstrated to exert a substantial influence on the composition of local pathogen and vector communities (Altizer et al. 2013; Clark et al. 2018; Gage et al. 2008). Consequently, habitat climatic conditions may influence the evolution of the immune system by influencing the distribution of pathogens and the dynamics of host‐pathogen interactions (O'Connor et al. 2020). Understanding the molecular adaptability of animal immune genes to their environments is of significant importance, as it may help elucidate immune defense mechanisms across diverse habitats and contribute to our comprehension of global biodiversity patterns.

Toll‐like receptors (TLRs) represent a family of evolutionarily conserved transmembrane pattern recognition receptors that are found in animals (Akira and Takeda 2004; Leulier and Lemaitre 2008). TLRs are pivotal in initiating innate immune responses and are capable of recognizing pathogen‐associated molecular patterns (PAMPs) from microorganisms, including viruses, bacteria, and fungi (Fitzgerald and Kagan 2020; Jin and Lee 2008). After binding to pathogens, TLRs initiate intracellular signaling cascades, promoting the production of pro‐inflammatory cytokines and subsequently triggering an inflammatory response in infected host tissues to protect the host from invasive microorganisms (Kawai and Akira 2010; Levy et al. 2020). The general structure of TLRs is strictly conserved and composed of three structural domains. The extracellular domain (ECD) consists of multiple leucine‐rich repeat sequences (LRRs) that are involved in pathogen recognition (Jin et al. 2007). The ECD is followed by a short transmembrane domain (TM) and an intracellular domain (ICD). The latter contains the Toll/Interleukin‐1 Receptor (TIR) domain, which is responsible for TLR signaling (Barton and Medzhitov 2003; Fitzgerald and Kagan 2020; Kawai and Akira 2010).

The TLR family is comprised of 10–12 members in most mammals, with 13 members being found in rodents (Roach et al. 2005; Su et al. 2024). Based on their cellular localization and recognized PAMPs, TLRs can be classified into two major functional groups (Akira et al. 2006). The first group includes viral TLRs (TLR3, TLR7, TLR8, and TLR9), which are primarily located on the membranes of endosomal compartments. The second group consists of non‐viral TLRs (TLR1, TLR2, TLR4, TLR5, TLR6, and TLR10), which are predominantly located on cell membranes (Barreiro et al. 2009; Kawai and Akira 2010; Leulier and Lemaitre 2008; Tassia et al. 2017). Moreover, TLRs can be categorized into six distinct subfamilies according to their structure and phylogenetic position (Roach et al. 2005). TLRs represent a prominent class of pattern recognition receptors that have undergone particularly extensive functional research in the context of the innate immunity of vertebrates (Leulier and Lemaitre 2008). Research has found that in both humans and experimental mice, genetic polymorphisms of *TLR* genes are associated with susceptibility to multiple infectious diseases and resistance to various pathogenic factors (Cook et al. 2004; Mukherjee et al. 2019; Schröder et al. 2005; Skevaki et al. 2015; Wang et al. 2014). For instance, a single nucleotide polymorphism (SNP) in the *TLR2* gene has been associated with an increased risk of tuberculosis, cytomegalovirus infection, and borreliosis (Skevaki et al. 2015), while genetic variations in the *TLR8* gene have been linked to human immunodeficiency virus (HIV) diseases, pulmonary tuberculosis, and hepatitis C virus (HCV) infections (Davila et al. 2008; Oh et al. 2008; Wang et al. 2014). In addition, associations have been identified between the polymorphic loci of *TLR* genes and the infection of wild rodents by pathogens. For example, the *TLR2* polymorphism of *Myodes glareolus* has been shown to be significantly correlated with Borrelia infection status, while genetic variations of *TLR2* and *TLR8* in *Rattus norvegicus* have been found to be associated with *Bartonella* and HEV infections (Cornetti et al. 2018; Su et al. 2022; Tschirren et al. 2013).

As TLRs are located at the interface between the host and the environment, coevolution with pathogens can promote the diversification of *TLR* genes through specific adaptation to ligand variations (Barreiro et al. 2009; Sironi et al. 2015). This makes them an ideal model for the study of the basic characteristics of host‐pathogen molecular coevolution. Numerous studies have demonstrated that purifying selection exerts a predominant effect on the evolution of *TLR* genes, thereby maintaining functional constraints (Liu et al. 2020). However, adaptive evolution of *TLR* genes has been observed in various vertebrates, including birds, cetaceans, and primates (Alcaide and Edwards 2011; Velová et al. 2018; Wlasiuk and Nachman 2010; Xu et al. 2019). Certain regions of *TLR* genes in these species exhibit evolutionary plasticity, potentially shaped by pathogen‐mediated adaptive evolution. Furthermore, the genetic diversity and evolutionary rates of non‐viral *TLR* genes are higher than those of viral *TLR* genes (Barreiro et al. 2009; Liu et al. 2020). Studies on pathogens have demonstrated significant structural variations in PAMPs among bacterial species (Kang and Lee 2011; Maeshima and Fernandez 2013). Non‐viral *TLR*s can recognize a diverse array of ligands with varying structures and exhibit greater redundancy in bacterial recognition (Vinkler et al. 2014; Wlasiuk and Nachman 2010). In contrast, viral *TLR*s are subject to substantial selection constraints and play a critical non‐redundant role in host survival (Liu et al. 2020). Notably, the distinct selection patterns between viral and non‐viral *TLR* genes have been observed across various mammalian groups, including primates and carnivores (Barreiro et al. 2009; Liu et al. 2017; Wlasiuk and Nachman 2010).

Rodents represent a highly species‐rich and widespread group of mammals, inhabiting a range of climate zones (Chen et al. 2023; Churakov et al. 2010). These animals act as hosts for various pathogenic organisms, and several species, such as rats and mice, have become firmly associated with human environments, thereby posing potential public health risks (Han et al. 2015; Kváč et al. 2013; Luis et al. 2013). Therefore, rodents are ideal subjects for studying the evolution of the immune system, and their *TLR* genes polymorphisms and molecular adaptability have attracted attention (Fornůsková et al. 2013; Tschirren et al. 2011; Kloch et al. 2018). Research has demonstrated that the evolution of *TLR* genes in rodents is also predominantly driven by purifying selection, although instances of positive selection have also been identified. The number of positive selection sites in *TLR* genes varies among different rodent species. Furthermore, non‐viral *TLR* genes also exhibit a greater number of positively selected sites than viral *TLR* genes (Fornůsková et al. 2013; Su et al. 2022, 2024; Tschirren et al. 2011; Turner et al. 2012). However, current research on the evolution of *TLR* genes in rodents primarily focuses on the adaptation of certain species to infectious pathogens, and the identification of positively selected sites. Understanding of how TLR genes are associated with ecological variables at a macroevolutionary scale remains limited. In addition, host species that share close genetic relationships are frequently colonized by closely related microbial communities (Kropáčková et al. 2017). This close relationship between host and pathogen often results in continuous co‐adaptation that corresponds to phylogenetic patterns. Therefore, analyses incorporating host ecological variables and phylogenetic relationships may yield insights into potential factors associated with the molecular evolution of *TLR* genes. To investigate the association of climatic factors with the evolutionary rate of the *TLR* gene family in rodents, this study initially calculated the ratio of nonsynonymous to synonymous substitution rates (*ω* = dN/dS) for *TLR* genes across 42 rodent species. Subsequently, comparative phylogenetic analyses including phylogenetic analysis of variance (phyANOVA) and phylogenetic generalized least squares (PGLS) were employed to evaluate the relationship between *ω* and climatic factors, while also considering phylogenetic independence. Our research hypothesized that certain *TLR* genes in rodents exhibit specific correlations with climatic factors. These results may offer exploratory insights into the evolutionary patterns of *TLR* genes and the potential links between environmental variation and host‐pathogen interactions.

## 2 Materials and Methods

### 2.1 Species Sample and Climate Dataset

Increased dispersal capacity and a broader geographical distribution are associated with greater risk of pathogen spread and infection, which poses a significant challenge to animals exposed to various pathogens (Altizer et al. 2011; O'Connor et al. 2018). In order to investigate the association between distinct climatic factors and the evolution of *TLR* genes, we selected 42 rodent species for which whole‐genome data are available and whose natural distributions are restricted to specific zoogeographic regions. Initially, 119 available *TLR* gene sequences from 15 rodent species were obtained through the NCBI GenBank database (https://www.ncbi.nlm.nih.gov, Table S1), which constituted the seed set. Subsequent to this, the genomic data for the additional species included in the study was obtained from the NCBI in order to create a local database. The identification of *TLR* genes was facilitated by utilizing BLASTN, employing the seed sequences as queries in a comprehensive whole‐genome scan of the constructed local genomic database. All candidate TLR homologs identified by BLASTN were filtered using a stringent E‐value threshold (< 1e‐5) and sequence identity criteria (> 70%) in order to reduce the number of false‐positive hits. The detailed genome information and scaffold information of newly identified *TLR* gene sequences were listed in Table S1.

The longitude and latitude data for 42 rodents were obtained from the Global Biodiversity Information Facility (GBIF) (Global Biodiversity Information Facility 2022). After removing redundant and erroneous records, we further addressed potential spatial biases by sorting the coordinates separately by longitude and latitude and excluding the highest and lowest 5% of records for each axis to remove spatial outliers. The dataset under consideration includes species and family names, geographic distribution details, and dynamic traits for each species. Geographical coordinates were used to access climate data from the WorldClim database (https://www.worldclim.org) at a 2.5 arc‐min resolution. The final set of 19 climate factors for each species was calculated using the capping method and subsequently averaged. Pearson correlation analysis was conducted on all environmental variables, and then 10 environmental variables with relatively low correlations (Pearson *r* < 0.8) were chosen for further analyses: Annual Mean Temperature (BIO1), Mean Diurnal Range (BIO2), Temperature Seasonality (BIO4), Max Temperature of Warmest Month (BIO5), Mean Temperature of Wettest Quarter (BIO8), Precipitation of Wettest Month (BIO13), Precipitation of Driest Month (BIO14), Precipitation Seasonality (BIO15), Precipitation of Warmest Quarter (BIO18), and Precipitation of Coldest Quarter (BIO19). Based on these climate variables, the rodents were categorized into three groups, arranged from low to high, for subsequent analysis of variance.

### 2.2 Phylogenetic and Molecular Evolutionary Analyses

The Bayesian Inference (BI) method was utilized for phylogenetic analysis in the construction of gene tree to further classify each *TLR* gene to its corresponding cluster. Initially, all identified *TLR* gene sequences were aligned using MAFFT within PhyloSuite, employing the auto strategy and codon alignment mode (Katoh and Standley 2013; Zhang et al. 2020). Subsequently, the SYM + I + G model was identified as the best‐fitting substitution model using the ModelFinder. The Markov chain Monte Carlo (MCMC) chains were run for 1,000,000 generations, with a sampling frequency of one tree every 1000 generations during the Bayesian analysis (Ronquist et al. 2012). The convergence between the runs was evaluated by monitoring the average standard deviation of split frequencies (ASDSF). The analysis was considered convergent once the ASDSF dropped below the threshold of 0.05. The BI tree was visualized using the Interactive Tree of Life (ITOL) website (Letunic and Bork 2019).

To evaluate the evolutionary rates of *TLR* genes across species and to investigate their correlations with climatic factors, the branch model (free‐ratio versus one‐ratio model) within the CODEML program of PAML was utilized to calculate the *ω* ratio for each branch based on the phylogenetic tree generated from Upham et al. (2019) (Upham et al. 2019; Yang 2007). The likelihood ratio test (LRT) was employed to assess the statistical significance of the models, using a threshold of *p* ≤ 0.05 to establish significance. The ratio of *ω* is an indication of purifying selection when < 1, neutral evolution when = 1, and positive selection when > 1. All *ω* values were log‐transformed to improve normality for statistical comparison and regression analysis.

### 2.3 Phylogenetic Comparative Analysis

A range of standard statistical and regression analyses were conducted in order to explore the relationship between the evolutionary rates of *TLR* genes and various climatic factors. The statistical analyses employed a combination of phylogenetic analysis of variance (phyANOVA) and multiple comparison analysis (*t*‐test) to evaluate the *ω* values of rodents across different groups, categorized based on the aforementioned climatic classifications. The phyANOVA analyses were executed using the packages of ape, geiger and phytools in R (Harmon et al. 2008; Paradis et al. 2004; Revell 2012). Post hoc pairwise comparisons among climatic groups were performed when the overall phyANOVA was significant, and *p* values from pairwise comparisons were adjusted using Holm's method to control for multiple testing. Moreover, the phylogenetic generalized least squares (PGLS) regression analyses were employed to explore the potential correlation between the *ω* values and climatic factors. These analyses were conducted using the Caper package in R, with the phylogenetic signal *λ* assessed via the maximum likelihood method (ML), ranging from 0 to 1 (Orme et al. 2011). A value close to 0 indicates phylogenetic independence, while a value near 1 suggests strong phylogenetic dependence. For the analyses conducted, a time‐scaled phylogenetic tree was generated, incorporating 42 rodent species as the input tree file (Figure S1) (Upham et al. 2019). To account for multiple testing across different TLR genes and climatic variables, false discovery rate (FDR) correction was applied to the main *p* values obtained from both PGLS and phyANOVA analyses using the Benjamini–Hochberg procedure.

## 3 Results

### 3.1 *TLR* Genes Identification and Climate Dataset Collection

To investigate the association between climate factors and the evolutionary rate of *TLR* genes, while minimizing the influence of heightened pathogen infection risk associated with larger distribution areas, this study focused on 42 rodent species inhabiting smaller distribution areas. These species are distributed across various climatic zones, including tropical rainforests, temperate grasslands, and frigid tundra. We initially retrieved annotated *TLR* gene sequences from the GenBank database. For any missing or inaccurate genes, local BLASTN was used to predict gene models based on the alignment sequences. Given the substantial deficiency of the identified *TLR10–TLR13* genes, we focused solely on the *TLR1–TLR9* genes for subsequent analyses, resulting in a total of 367 gene sequences. Additionally, we gathered current distribution and climate data for 42 rodent species, which have been processed and were presented in Table S2.

### 3.2 Phylogenetic Tree of All *TLR* Genes and Calculation of *ω* Value

**FIGURE 1:**
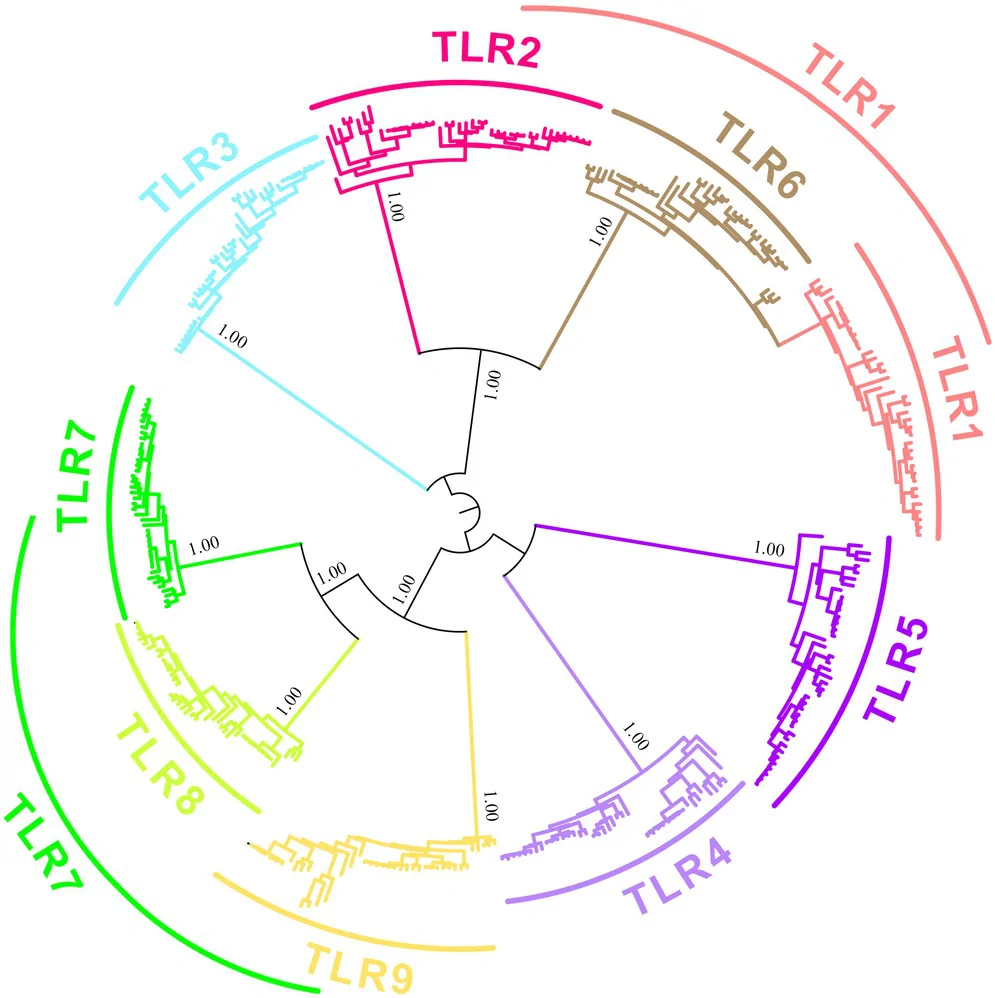
The BI tree of 42 rodent *TLR1*–*TLR9*, illustrating the division of the gene family into five subfamilies.

The phylogenetic analysis indicated that the *TLR1*–*TLR9* can be distinctly categorized into five independent *TLR* subfamilies. Specifically, *TLR1*, *TLR2*, and *TLR6* were found to cluster to form the *TLR1* subfamily, while *TLR7*, *TLR8*, and *TLR9* were identified as components of the *TLR7* subfamily. Furthermore, *TLR3*, *TLR4*, and *TLR5* each represented three independent subfamilies (Figure 1). This classification model exhibited a complete alignment with the previously established *TLR* gene family classification system (Takeda et al. 2003). The *TLR* genes across all rodents were grouped into independent specific lineages characterized by relatively high Bayesian posterior probabilities (Figure S2). It is noteworthy that *TLR1* and *TLR6* were grouped together with a relatively high posterior probability; however, certain species sequences exhibited considerable cross‐mixing within the clustering lineage. This indicated that the *TLR1* and *TLR6* genes of these rodents exhibit a higher degree of sequence similarity, which may be associated with the function of their expression products in forming heterodimers with TLR2 to recognize pathogens (Takeda et al. 2003; Takeuchi and Akira 2010). Each *TLR* gene lineage was generally found to be distinctly differentiated into five branches, corresponding to Cricetidae, Muridae, Sciuridae, Heteromyidae+Geomyidae, and Erethizontidae+Bathyergidae. Notably, the topological relationships of each taxonomic unit within the *TLR1* and *TLR8* lineages aligned closely with the established phylogeny of rodents (Fabre et al. 2012; Huchon et al. 2002). In contrast, the *TLR4* and *TLR7* lineages placed the Heteromyidae+Geomyidae branch at the base of the topological structure. The internal topological structures of the remaining *TLR* gene branches exhibited certain differences, and the posterior probability support levels for the related branches were relatively low (Figure S2). Overall, the *TLR* genes of the 42 rodents identified can be considered to be accurate and are suitable for subsequent analyses.

The branch models were then employed to calculate the evolutionary rates of *TLR* genes across the rodent species. The *TLR* genes evaluated using the one‐ratio model exhibited relatively low *ω* values, ranging from 0.1795 to 0.4180 (Table S3). Furthermore, the *ω* values for non‐viral *TLR* genes (*TLR1*, *TLR2*, *TLR4*, *TLR5*, and *TLR6*) were significantly higher than those for viral *TLR* genes (*TLR3*, *TLR7*, *TLR8*, and *TLR9*) (*p* < 0.05). This finding suggests that rodent *TLR* genes are predominantly under purifying selection, whereas viral *TLR* genes experience a more pronounced selective pressure. With the exception of the *TLR5* gene, the LRT between the free‐ratio model and the one‐ratio model was significant (*p* < 0.05), indicating that the free‐ratio model fitted the data better. Therefore, the *ω* values of each branch from these eight genes were used for subsequent comparisons and correlation analyses (Table S4). Because *TLR5* did not show significant branch‐specific variation under the free‐ratio model, it was excluded from the subsequent phyANOVA and PGLS analyses.

### 3.3 Statistical Comparisons of *ω* Ratio and Selection of Different Groups

**FIGURE 2:**
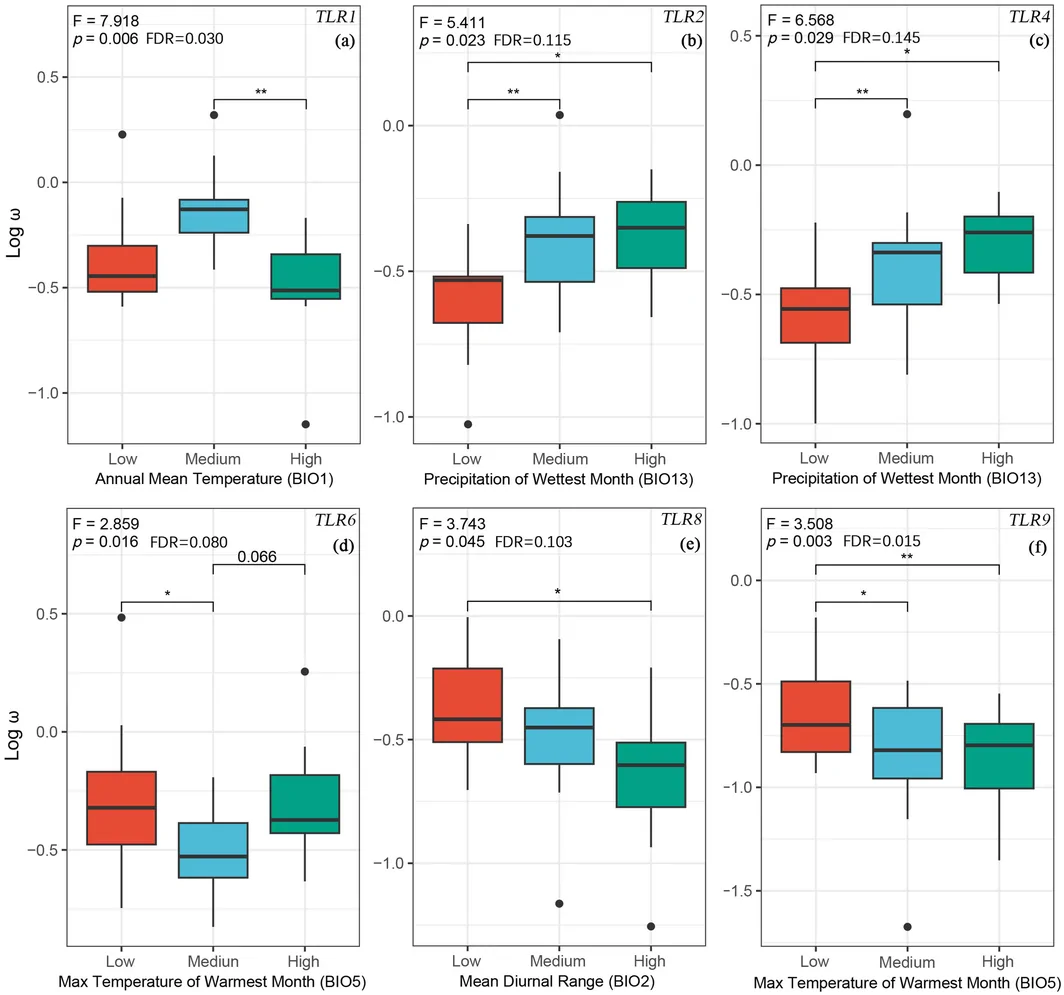
Statistical comparisons of *ω* values grouped by climatic factors. X axis: Categories of climatic factors. Y axis: *ω* values of *TLR* genes after log transformation. Species were classified into three climatic categories according to the rankings of climatic factors. The *F*‐value and *p*‐value located in the top left corner of the boxplot were extracted from results of phyANOVA. Pairwise comparisons were performed through *t*‐test method, * and ** above horizontal lines represent statistically significant (*p* < 0.05, *p* < 0.01).

To investigate the relationship between the evolutionary rates of *TLR* genes and various climatic factors, we employed phyANOVA, which accounts for evolutionary relationships, along with multiple comparison methods to compare the *ω* values for *TLR* genes in rodents across different climatic categories (Low/Medium/High). After FDR correction, two phyANOVA associations remained significant. In general, the *ω* values for *TLR1* exhibited significant differences among groups based on Annual Mean Temperature (BIO1) (*F*‐value = 7.918, *p* = 0.006, *FDR* = 0.030) (Figure 2a, Table S5). The Medium BIO1 Group displayed the highest *ω* values for *TLR1*, significantly exceeding those of the High BIO1 Group. Additionally, nominally significant differences in *ω* values for *TLR2* and *TLR4* were noted among groups based on the Precipitation of Wettest Month (BIO13) (*TLR2*, *F*‐value = 5.411, *p* = 0.023, *FDR* = 0.115; *TLR4*, *F*‐value = 6.568, *p* = 0.029, *FDR* = 0.145) (Figure 2b,c). The *ω* values for these two genes in the Low BIO13 Group were significantly lower than those in the other two groups. However, these two associations did not remain significant after FDR correction and should therefore be interpreted cautiously. Furthermore, the *ω* values for *TLR6* and *TLR9* showed significant differences among groups categorized by the Max Temperature of Warmest Month (BIO5) (*TLR6*, *F*‐value = 2.859, *p* = 0.016, *FDR* = 0.080; *TLR9*, *F*‐value = 3.508, *p* = 0.003, *FDR* = 0.015) (Figure 2d,f). Multiple comparisons indicated that the *ω* values for these *TLR* genes exhibited distinct relationships with this climatic factor. Specifically, the *ω* values for *TLR9* in the Low BIO5 Group were significantly greater than those in the other two groups. Conversely, the Medium BIO5 Group exhibited the lowest *ω* values for *TLR6*, which were significantly lower than those in the Low BIO5 Group and slightly lower than those in the High BIO5 Group. After FDR correction, the association between TLR9 and BIO5 remained significant, whereas TLR6 under BIO5 showed only a marginal trend. Finally, the *ω* values for *TLR8* revealed significant differences among groups based on Mean Diurnal Range (BIO2) (*F*‐value = 3.743, *p* = 0.045, *FDR* = 0.103) (Figure 2e). The Low BIO2 Group recorded the highest *ω* values for *TLR8*, which were significantly greater than those in the High BIO2 Group. However, this association did not remain significant after FDR correction. Overall, the phyANOVA results suggest that some *TLR* genes show climate‐associated group differences in evolutionary rates, but only *TLR1* under BIO1 classification and *TLR9* under BIO5 classification remained significant after FDR correction. Therefore, the remaining nominal associations only should be interpreted as suggestive.

### 3.4 Correlation Between Climatic Factors and *ω* Value

**FIGURE 3:**
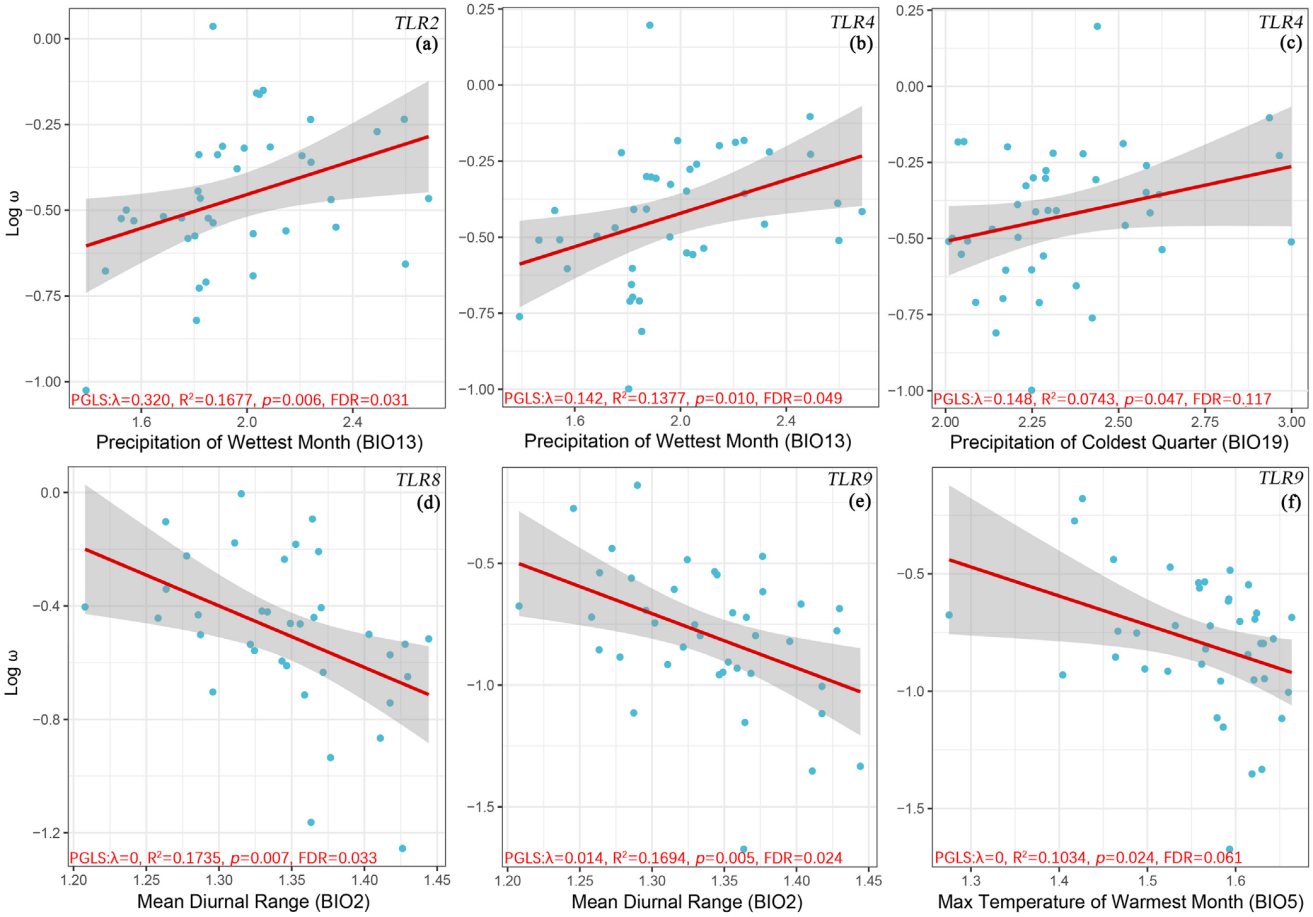
Regression analyses between *ω* values and climatic factors by PGLS. The solid lines represent the regressions from the PGLS methods with the slope and intercept values displayed.

We further conducted PGLS regression analyses in order to investigate the relationship between climatic factors and the evolutionary rates of *TLR* genes. After FDR correction, four PGLS associations remained significant. The results indicated that the *ω* values for non‐viral *TLR* genes, specifically *TLR2* and *TLR4*, exhibited significant correlations with the Precipitation of Wettest Month (BIO13) (Figure 3a,b, Table 1). Additionally, the *ω* values for *TLR4* were also found to be nominally associated with the Precipitation of Coldest Quarter (BIO19) (Figure 3c). However, this association did not remain significant after FDR correction. The correlation slope suggested that the *ω* values for both *TLR2* and *TLR4* were positively correlated with these precipitation‐related climatic factors. Furthermore, PGLS analyses revealed that the *ω* values for viral *TLR* genes, including *TLR8* and *TLR9*, displayed significant negative correlations with the Mean Diurnal Range (BIO2) (Figure 3d,e). The *ω* values for *TLR9* were also nominally associated with the Maximum Temperature of Warmest Month (BIO5) (Figure 3f). This association showed a marginal trend after FDR correction but did not meet the FDR < 0.05 threshold. The detected phylogenetic signal (*λ*) ranged from 0 to 0.320, indicating varying degrees of phylogenetic constraints on different genes (Table 1). Notably, the *λ* values for viral *TLR* genes were lower than those for non‐viral *TLR* genes, often approaching 0, suggesting that the evolution of viral *TLR* genes was largely unaffected by phylogenetic factors. However, because the *R* ^2^ values of the PGLS models were generally low, with the highest value being 0.1735, climatic variables explained only a limited proportion of variation in TLR evolutionary rates. Overall, significant differences were observed in the response patterns of different *TLR* genes evolution to climatic factors.

**TABLE 1:** Significant results of PGLS analyses for *ω* values with climatic factors.

Response variable	Predictor variable	Slope	*λ*	*R* ^2^	*p*	*FDR*
*TLR2*	Precipitation of Wettest Month (BIO13)	0.28832	0.320	0.1677	0.006	0.031*
*TLR4*	Precipitation of Wettest Month (BIO13)	0.27832	0.142	0.1377	0.010	0.049*
*TLR4*	Precipitation of Coldest Quarter (BIO19)	0.27008	0.148	0.0743	0.047	0.117
*TLR8*	Mean Diurnal Range (BIO2)	−2.17046	0	0.1735	0.007	0.033*
*TLR9*	Mean Diurnal Range (BIO2)	−2.23326	0.014	0.1694	0.005	0.024*
*TLR9*	Maximum Temperature of Warmest Month (BIO5)	−1.23778	0	0.1034	0.024	0.061

* *q* < 0.05.

## 4 Discussion

As crucial pattern recognition receptors within the innate immune system, previous research indicates that the evolution of *TLR* genes is influenced by pathogen stress. However, climatic factors, such as temperature and precipitation, have been identified as pivotal ecological variables in influencing the distribution, transmission rate, and pathogenicity of pathogens (Altizer et al. 2006; Haines et al. 2006; Patz et al. 2005). The regulatory effects of these climatic factors on the evolutionary rate of *TLR* genes have not been systematically investigated. In this research, we focused on the relationship between the evolutionary rates of *TLR* genes in rodents and climatic factors. Our results revealed partial and gene‐specific associations between climatic variables and TLR evolutionary rates. Temperature‐related climatic factors (BIO1, BIO2, BIO5) were associated with variation in the evolutionary rates of *TLR1*, *TLR8*, and *TLR9*, while precipitation‐related factors (BIO13) were primarily associated with *TLR2* and *TLR4*. Therefore, our findings provide exploratory evidence for climate‐driven evolution of rodent TLR genes.

### 4.1 Elevated Precipitation May Accelerate the Evolution of *TLR* Genes

In this study we found that the *ω* values for *TLR2* and *TLR4* in rodents exhibited a high degree of positive correlation with Precipitation of Wettest Month (BIO13) (Figure 3a,b). These results suggest that high‐precipitation environments may be associated with elevated evolutionary rates of *TLR2* and *TLR4*. TLR2 has an extremely wide recognition range and typically forms heterodimers with TLR1 or TLR6. The primary function of this system is the identification of components of Gram‐positive bacteria, as well as the recognition of microorganisms such as fungi (Hamilos 2014; Takeuchi et al. 1999). TLR4 is the main receptor for recognizing lipopolysaccharide (LPS) of Gram‐negative bacteria (Mukherjee et al. 2016; Sun et al. 2012). BIO13 serves as crucial metrics for assessing environmental humidity. Regions with abundant rainfall, particularly during specific seasons, exhibit notably elevated humidity compared to other areas. This moist setting creates optimal circumstances for the proliferation and dissemination of various pathogens, including Gram‐negative and Gram‐positive bacteria, as well as fungi (Guernier et al. 2004). The increase in precipitation directly leads to an increase in the diversity and abundance of the environmental microbiome. Therefore, species inhabiting humid environments may be exposed to a broader spectrum of pathogens (Keesing et al. 2006; Yang et al. 2012).

According to the Red Queen Hypothesis, hosts and pathogens maintain a dynamic equilibrium of defense and offense through coevolution (Siddle and Quintana‐Murci 2014). Hosts primarily enhance their defensive recognition capabilities by increasing the diversity or adaptability of immune receptors, while pathogens evolve strategies to resist or evade these defenses (Siddle and Quintana‐Murci 2014; Su et al. 2024; Tschirren et al. 2013). Studies have shown that resident birds inhabiting humid regions, particularly in tropical Africa, exhibit greater MHC‐I diversity than those residing in arid areas, and these birds in humid tropical environments have developed a more robust capacity to recognize pathogens (O'Connor et al. 2020). Rodents inhabiting regions with high precipitation levels are exposed to environments characterized by a high abundance of pathogens. The elevated *ω* values observed in *TLR2* and *TLR4* may reflect stronger or more diverse pathogen‐mediated selective pressures in humid environments. This observation is consistent with the findings of previous studies, which have indicated that an increased diversity of environmental microorganisms is associated with a faster rate of evolution of immune genes (Brockhurst et al. 2014; Eizaguirre et al. 2012). Consequently, precipitation variables may represent indirect ecological proxies associated with variation in the evolutionary rates of *TLR2* and *TLR4*.

### 4.2 Temperature Affects the Different Evolutionary Patterns of *TLR* Genes

Temperature serves as a critical environmental factor influencing the geographical distribution, abundance, diversity, and population structure of pathogens, particularly bacteria and fungi (Altizer et al. 2006, 2013; Garcia‐Solache and Casadevall 2010). Various *TLR* genes are tasked with detecting distinct types of pathogens, and these pathogens exhibit varying responses to temperature changes (Garcia‐Solache and Casadevall 2010; Harvell et al. 2002; Kawai and Akira 2010). In contrast to the findings related to precipitation factors, our analysis revealed a significant negative correlation between the *ω* values for *TLR8* and *TLR9* and Mean Diurnal Range (BIO2) (Figure 3d,e). Additionally, a marginal association was detected between *TLR9* and Max Temperature of Warmest Month (BIO5), although this relationship did not remain significant after FDR correction in PGLS analysis (Figures 2f and 3f). TLR8 and TLR9 are located on the endosomal membrane of cells, where they specifically recognize single‐stranded RNA (ssRNA) and unmethylated CpG DNA, respectively (Jiménez‐Dalmaroni et al. 2016). Their functions are highly dependent on the structural conservation of the receptor (Barton and Kagan 2009; Krieg 2002). We observed lower evolutionary rates in viral *TLR*s (including *TLR8* and *TLR9*), indicating that these receptors are under stronger purifying selection, which preserves the stability of their core functions in recognizing pathogens. Several studies have highlighted that the ligand recognition sites of endosomal *TLR*s, such as *TLR7*, *TLR8*, and *TLR9*, are highly conserved across various groups, with changes primarily occurring at specific sites and often linked to lineage‐specific pathogens (Georgel et al. 2009; Heni et al. 2020; Roach et al. 2013; Wlasiuk and Nachman 2010).

High BIO2 values indicate more intense temperature fluctuations. The significant fluctuations may impair the reproductive capacity of pathogens, thereby affecting their survival in the environment (Tang 2009). For example, the ssRNA of the influenza virus can be recognized by TLR8. Only under stable low‐temperature conditions, the envelope protein of the influenza virus is less prone to conformational changes or denaturation, which greatly enhances the virus's stability in vitro (Lowen et al. 2007). In a high BIO2 environment, the diversity of pathogen communities encountered by the host may be relatively low, resulting in reduced pathogen pressure (Altizer et al. 2006). Furthermore, previous research has shown that maintaining the immune system and initiating immune responses require substantial energy. Natural selection does not always favor the strongest immune responses. Consequently, under conditions of intense environmental fluctuations, hosts may trade off immune capacity to prioritize reproduction and growth (Lazzaro and Little 2009). We hypothesized that increased temperature fluctuations may have contributed to a reduction in the positive selection pressure among *TLR8* and *TLR9* in a high BIO2 environment, resulting in the observed negative correlation between the *ω* values and BIO2. High BIO5 represents an extreme high‐temperature pressure, similar to BIO2, which may restrict the diversity and transmission of pathogens recognized by TLR9 (Altizer et al. 2006; Tang 2009). This limitation significantly reduces the infection pressure experienced by the host and diminishes the selection pressure on *TLR9*. Additionally, under high‐temperature stress, the organism enters a state of heat stress, making it susceptible to the generation of endogenous damage‐associated molecular patterns (DAMPs) (Iba et al. 2025). If *TLR9* evolves too rapidly, resulting in altered sensitivity, it may misidentify its own DNA as an exogenous pathogen during heat stress, potentially leading to severe inflammation (Barton and Kagan 2009). Consequently, the conservation of *TLR9* under extreme high‐temperature conditions may be essential for preventing autoimmune responses triggered by the release of endogenous DNA during heat stress. Overall, the diversity of pathogens in environments characterized by significant temperature fluctuations and extreme high temperatures is strongly influenced. We speculated that the specific pathogen pressure encountered by the host may become unique and diminished, resulting in *TLR* genes undergoing intense purifying selection.

The phyANOVA analyses indicated that *TLR1* and *TLR6* showed distinct group‐level patterns with temperature‐related variables (Figure 2a,d). In particular, the *ω* values for *TLR1* peaked in the medium BIO1 group, while the *ω* values for *TLR6* were at their lowest in the medium BIO5 group. However, the association between *TLR6* and BIO5 was only marginally significant after FDR correction. TLR1 and TLR6 form heterodimers with TLR2 (Takeda et al. 2003; Takeuchi and Akira 2010). The TLR1/TLR2 complex primarily recognizes triacyl‐lipopeptides, which are predominantly derived from bacteria and mycobacteria (Jin et al. 2007). This complex is situated on the cell membrane and serves as one of the initial lines of defense against extracellular pathogens. In contrast, the TLR6/TLR2 complex predominantly recognizes diacyl‐lipopeptides, which are primarily sourced from certain bacteria, mycoplasma, and fungi (Takeda et al. 2003). There exists both functional overlap and a division of labor between TLR1 and TLR6 (Farhat et al. 2008). For *TLR1*, environments with moderate annual average temperatures may represent hotspots for pathogen diversity and transmission, characterized by a wide array of lipopeptide variations that drive the rapid evolution of the TLR1 receptor binding domain, thereby preserving its recognition capability (Altizer et al. 2006; Kawai and Akira 2010). This finding was consistent with the prevailing ecological understanding that mid‐latitude or temperate regions characteristically exhibit elevated microbial community complexity (Dlugosch et al. 2022). Consequently, the *TLR1* of rodents inhabiting these regions exhibited an elevated evolutionary rate at moderate temperatures. The notable differences in the evolutionary rate of *TLR6* in response to temperature may be attributed to variations in temperature factors. The BIO1 reflects the overall thermal conditions and influences pathogen diversity throughout the year. Conversely, the BIO5 indicates extreme high temperatures, which may be selected for specific heat‐resistant pathogens. Additionally, differences exist in the pathogen recognition spectra of TLR1 and TLR6. We proposed that the lower extreme temperature represents the most favorable ecological niche for the specific pathogen recognized by TLR6. In this context, the host experiences heightened pathogen infection pressure, which fosters relatively strong positive selection for *TLR6*. As extreme high temperatures rise further, the infection pressure from pathogens diminishes, thereby reducing the positive selection pressure on *TLR6* and resulting in a decreased evolutionary rate. However, at the highest extreme temperatures, the evolutionary rate of *TLR6* exhibited a slight increase, potentially due to the host's diminished capacity to recognize pathogens via TLR6 under extreme thermal conditions, which leads to a relaxation of selection pressure. We hypothesized that as the extreme temperatures increase from low to high, the *TLR6* has successively undergone positive selection due to pathogen pressure, strong purifying selection, and then relaxation of selection pressure. Overall, this finding suggested that the response of different pathogen groups to temperature may significantly influence the evolutionary patterns of host immune genes.

It is essential to emphasize that our analyses are of a correlational nature, and do not establish any form of direct causality between climatic variables and *TLR* evolutionary rates. Climatic factors likely influence immune gene evolution indirectly through changes in pathogen diversity, ecological exposure, and host population structure. Furthermore, the absence of direct pathogen diversity measurements limits our ability to explicitly link environmental variation to selective pressures acting on *TLR* genes. It is imperative that future studies integrating metagenomic pathogen data and experimental validation are conducted in order to confirm these hypotheses.

## 5 Conclusions

This study systematically investigated the associations between climatic factors and the evolutionary rates of *TLR* genes across 42 rodent species, providing an exploratory perspective on potential environmental influences on innate immune gene evolution. Our results suggest that climatic factors may be correlated with the evolutionary dynamics of *TLR* genes in rodents, with distinct response patterns among TLR subfamilies that likely reflect their functional specialization. The increased precipitation‐related factors (BIO13) were found to be positively associated with the evolution of *TLR2* and *TLR4*, whereas rising temperature‐related factors (BIO2) showed negative correlations with the evolutionary rates of *TLR8* and *TLR9*. We hypothesized that these observed correlations may reflect indirect effects of climatic conditions on host‐pathogen interactions and environmental pathogen communities. The results provide preliminary and exploratory evidence for environmental influences on immune gene evolution.

## Author Contributions

**Chao Zhao:** conceptualization (equal), data curation (equal), methodology (equal), project administration (equal), visualization (equal), writing – original draft (equal). **Tian Xia:** data curation (equal), methodology (equal), project administration (equal). **Guangshuai Liu:** conceptualization (equal), methodology (equal), project administration (equal), validation (equal), writing – review and editing (equal). **Zhao Liu:** formal analysis (equal). **Xinglei Ding:** formal analysis (equal), software (equal). **Shuo Dai:** data curation (equal), formal analysis (equal), resources (equal). **Honghai Zhang:** conceptualization (equal), funding acquisition (equal), methodology (equal), writing – review and editing (equal).

## Conflicts of Interest

The authors declare no conflicts of interest.

## Supporting information


**Figure S1:** The time‐scaled phylogenetic tree incorporating 42 rodent species generated from Upham et al. (2019).

**Figure S2:** The BI tree of 42 rodent *TLR1*–*TLR9*, with Bayesian posterior probabilities were displated.

**Table S1:** Species, gene accession number or scaffold information for TLR genes in this study.

**Table S2:** Climate data sets.

**Table S3:** Likelihood ratio tests of branch models for the TLR genes.

**Table S4:** The *ω* values of each rodent calculate by free‐ratio model.

**Table S5:** The results of phyANOVA analysis.

**Table S6:** The original collected distribution coordinates and climate data for 42 rodent species.

## Data Availability

119 *TLR* gene sequences from 15 rodent species, along with 39 genomic datasets, were acquired from the NCBI GenBank database (https://www.ncbi.nlm.nih.gov). The gene accession numbers and detailed genomic information were presented in Table S1. The present distribution data for the 42 rodent species were sourced from the Global Biodiversity Information Facility (https://www.gbif.org/en/), and GBIF occurrence download 10.15468/dl.k7jcrf, 10.15468/dl.ngpcaj, 10.15468/dl.s8zvwu, and 10.15468/dl.gs4wrw. The climate data were sourced from the WorldClim database (http://www.worldclim.org) (Table S6).
