# Supplementary material for: Evolutionary Response Patterns of the Rodent TLR Genes to Climatic Factors
Source: Ecol Evol. 2026 Sep 1;16(9):e74271. doi: 10.1002/ece3.74271 (PMC13535156; doi:10.1002/ece3.74271)
Supplement: Supplementary file 2 [file ECE3-16-e74271-s007.pdf]

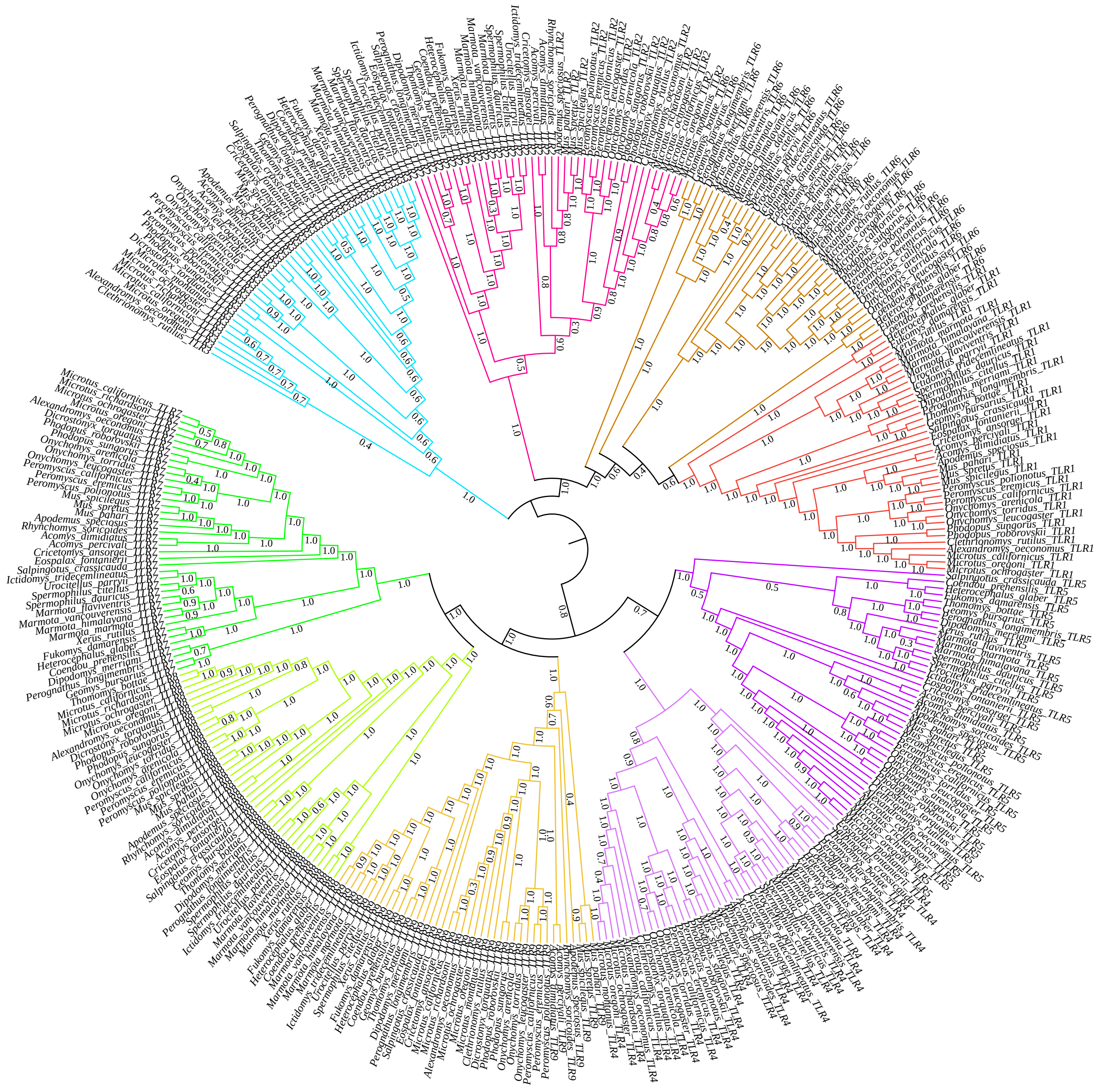

Figure S2 The BI tree of 42 rodent *TLR1-TLR9*, with Bayesian posterior probabilities were displated.
